# An implementation of the Gillespie algorithm for RNA kinetics with logarithmic time update

**DOI:** 10.1093/nar/gkv480

**Published:** 2015-05-18

**Authors:** Eric C. Dykeman

**Affiliations:** York Centre for Complex Systems Analysis, Department of Mathematics and Biology University of York, Deramore Lane, York, YO10 5GE, UK

## Abstract

In this paper I outline a fast method called KFOLD for implementing the Gillepie algorithm to stochastically sample the folding kinetics of an RNA molecule at single base-pair resolution. In the same fashion as the KINFOLD algorithm, which also uses the Gillespie algorithm to predict folding kinetics, KFOLD stochastically chooses a new RNA secondary structure state that is accessible from the current state by a single base-pair addition/deletion following the Gillespie procedure. However, unlike KINFOLD, the KFOLD algorithm utilizes the fact that many of the base-pair addition/deletion reactions and their corresponding rates do not change between each step in the algorithm. This allows KFOLD to achieve a substantial speed-up in the time required to compute a prediction of the folding pathway and, for a fixed number of base-pair moves, performs logarithmically with sequence size. This increase in speed opens up the possibility of studying the kinetics of much longer RNA sequences at single base-pair resolution while also allowing for the RNA folding statistics of smaller RNA sequences to be computed much more quickly.

## INTRODUCTION

The prediction of the secondary structure of an RNA molecule has been a heavily researched area for the last few decades and still remains one of the important challenges in biophysics and computational biology today. Computational techniques for predicting the minimum free energy secondary structure of an RNA based on the Turner rules for RNA base-pair stacking interactions (1) were pioneered by Zuker (2), and others (3) using a dynamic programming algorithm. Although these classic algorithms based on dynamic programming have become a powerful tool for predicting the minimum free energy structure of an RNA molecule as well as other sub-optimal structures, these methods provide only thermodynamic descriptions of the folding space and give little if no details on the kinetics of folding.

Plus-sense single-stranded RNA viruses (+ssRNA) provide a particularly salient example of the importance of the kinetics of RNA folding. The genomes of ssRNA viruses are under multiple evolutionary pressures to ensure that their genome is copied, that viral gene products are produced from the RNA genome (which also serves as an mRNA template) and that their genomes are selectively packaged during capsid assembly. Due to the multitude of functional roles that are required of ssRNA viral genomes during their life-cycle, RNA dynamics are expected to play a key role in regulating genome copying and viral protein production. For example, Simon and co-workers have recently demonstrated for Turnip Crinkle Virus (TCV), a small *T* = 3 plant virus, that the 3′ end of the viral genome transiently adopts different RNA structures which regulate translation by the RNA-dependent RNA polymerase (RdRp) (4). Similar regulatory features have also been seen in small self-replicating RNA fragments such as SV11 (5). In addition to regulating genome copying, the kinetics of RNA folding is also expected to play a crucial role during genome packaging in ssRNA viruses (6–10). Computational models which are capable of probing the kinetics of RNA refolding in large sequences (> 1000 nt) will present an opportunity to explore the regulatory and packaging roles of the viral genome in this class of viruses.

Kinetic folding algorithms have been previously developed to probe the dynamics of RNA and its alternative structures which may be only occupied transiently (11–14). A key issue with kinetic folding programs is the size of the RNA and length of folding time that can be simulated. For example, one of the first RNA kinetic folding algorithms, KINFOLD (11,12), models RNA kinetics at the most microscopic level possible, i.e. in terms of single base pair additions/deletions. However, currently it can only be used on small RNAs of around 50-100 nt due to both memory and computational time constraints. This problem has led to additional algorithms (13–19) that either model the kinetics of an RNA using a more macroscopic view in which the barriers between local minima in the energy landscape are first characterized then simulated using numerical integration (17,18) or fast-Fourier transforms (19). Additional methods have also allowed entire helices to elongate in a single reaction (20). Other algorithms have attempted to improve on the efficiency of the Gillespie procedure through the use of memorization of previous states (21). However, these methods are still limited to around a few 100 nt. Thus, current Gillespie algorithms which model RNA folding at single base-pair resolution still remain difficult to implement for longer sequences (> 1000 nt) due to computational time and computer memory constraints.

This paper introduces several computational strategies which allow the Gillespie algorithm (22) to be applied to the RNA kinetics problem with greater efficiency. The result is an algorithm in which the computational cost for calculating the transition to the next state performs logarithmically with the sequence length N. These new computational strategies open up new opportunities to examine the folding kinetics of large RNA sequences (> 1000 nt) at single base-pair resolution. First, a few current RNA kinetics algorithms are briefly discussed and the KINFOLD algorithm is introduced as a background of current state-of-the-art methods for computation of RNA folding kinetics at single base-pair resolution. Next, the KFOLD algorithm is introduced along with descriptions of the strategy used by KFOLD to achieve a substantial speed-up in computational time when compared to KINFOLD. Finally, the KFOLD algorithm is applied to several examples of RNA folding kinetics.

## MATERIALS AND METHODS

### Algorithms for RNA folding kinetics

A variety of work has been done on the problem of computing RNA folding kinetics resulting in two basic types of algorithms. The difference between the two types of algorithms relates to the degree of coarse-graining involved in the types of secondary structural rearrangements that occur when moving between RNA folding states. At one extreme, one could consider single base-pair additions/deletions when moving between RNA states. This type of algorithm would yield a more microscopic picture of RNA folding events and is the type that is implemented in the KINFOLD program (11,12). At the other end of the spectrum, one could consider much larger rearrangements of the base pairs in the RNA secondary structure when moving between different RNA states (13–19). In this strategy, the energy landscape is examined and local minima identified along with barriers (saddle points) between them. Both methods have their advantages and disadvantages. The advantage of considering single base-pair additions/deletions when moving between RNA states is that a reasonable estimate of the transition rate between the states *S*_*i*_ and *S*_*j*_ can be obtained from the free energy difference between the states Δ*G*_*ij*_ = *G*_*j*_ − *G*_*i*_ using:

(1) \begin{equation*} \frac{k_{ij}}{k_{ji}} = e^{-\beta \Delta G_{ij}} \end{equation*}

where *k*_*ij*_ denotes the transition rate from state *S*_*i*_ to state *S*_*j*_. An alternative method for estimating transition rates used by Flamm, called Kawasaki dynamics (23), uses a symmetric form of Equation (1) to uncouple *k*_*ij*_ from *k*_*ji*_, i.e.

(2) \begin{equation*} k_{ij} = k_0e^{-\beta \Delta G_{ij}/2}, \end{equation*}

where *k*_0_ is a pre-factor which can be adjusted to fit computational estimates of the folding times of RNAs to experimental measurements. Currently there are at least two disadvantages of Gillespie-type algorithms. The first is that the computational cost to compute a fixed number of changes to the secondary structure of the RNA in question, and thus its trajectory, is quite high and scales with the number of secondary structure neighbours *m*. The number of neighbours of an RNA secondary structure varies depending on its current fold and sequence but is related to sequence size *N* and is bounded from above by *m* = *N*^2^. The second is that many trajectories of the RNA are required to be computed in order to obtain reasonable statistics of the kinetics. Given these issues, single base-pair resolution methods have traditionally only been used on small RNAs with length less than around 100 nt.

Methods such as HIKINETICS (13,14), KINEFOLD (15,16) and others (17–19) are based on the second type of RNA folding algorithm and consider a more coarse-grained picture of RNA folding events, theoretically allowing for a faster computation of the RNA folding trajectories. For example, Senter *et al*. have used a fast-Fourier transform method to obtain fast approximate folding kinetics of RNAs (19), while Gies *et al*. have described a co-translational model of RNA folding (24). But these methods have the disadvantage that the transition rates between two RNA secondary structures differing by more than one base pair are not easily computed using Equation (1) above. This is due to the coarse-grained transition involving multiple single base pair transitions, with different barriers between these states. Estimating the transition rate in this regime is more complex and more computationally demanding and will be rate-limited based on the largest barrier encountered during the transition. Thus, the transition rate between local minima is not necessarily related to the energy difference between the local minimum. However, transition rates can still be estimated in these methods. For example, Huang *et al*. use a partition function method to estimate the kinetic transition rates between RNA structures which give reasonable kinetics for a variety of RNAs (13).

### The KFOLD algorithm

KFOLD implements the Gillespie algorithm (22) for computing RNA kinetics which is a type of continuous time Markov chain (CTMC) in which the probability of transitioning to the next state is independent of the previous state. The basic algorithm requires that the following two quantities be computed each time the RNA is moved to a new secondary structure: (i) the set of *m* neighbour structures *S*_*i*_ which differ from the current fold *S*_0_ by an appropriate move, e.g. a single base-pair addition/deletion; and (ii) the transition rate for moving from *S*_0_ to *S*_*i*_, *k*_0*i*_, using Equation (2) along with the total flux $\Phi = \sum _{i=1,m} k_{0i} = \sum _{i=1,m} k_0 e^{-\beta \Delta G_{0i}/2}$. These two computations are iteratively repeated to create a trajectory of structures that the RNA moves through in time. There are two major computational bottlenecks in a traditional implementation of this procedure. First, the time to compute the total flux Φ, which scales as O(*m*). And second, the time to compute the *m* secondary structures *S*_*i*_ which differ from *S*_0_ by an appropriate move along with the corresponding transition rate for the reaction *k*_0*i*_. When the difference between states *S*_*i*_ and *S*_0_ is a single base pair, then the energy difference between states can be calculated in O(1) time and the computational cost to compute neighbours and transition rates will also scale as O(*m*), giving a total scaling of the basic algorithm as O(*m*) per update of the RNAs secondary structure.

Although the KFOLD algorithm follows the same basic Gillespie protocol as KINFOLD, KFOLD achieves increased computational efficiency and a logarithmic cost per update of the RNA secondary structure by employing two strategies to reduce the computational costs described above. The first is to break up the problem of finding the structures *S*_*i*_ which neighbour a given RNA structure *S*_0_ into many smaller ones by finding neighbours of local secondary structures in the RNA. By doing this, only a small portion of the neighbour list will need to be updated between steps, decreasing the cost of calculating neighbours dramatically to approximately O(1). The second strategy uses a partial sum table to (i) compute the total flux Φ and, (ii) choose one of the reactions to fire in order Log_2_(*N*) time. The result of these two changes is a Gillespie algorithm for RNA kinetics in which the computational cost for computing updates to the secondary structure performs logarithmically with sequence size *N*. These two methods are discussed in the following sections.

#### Computing neighbouring structures in KFOLD

**Figure 1.**
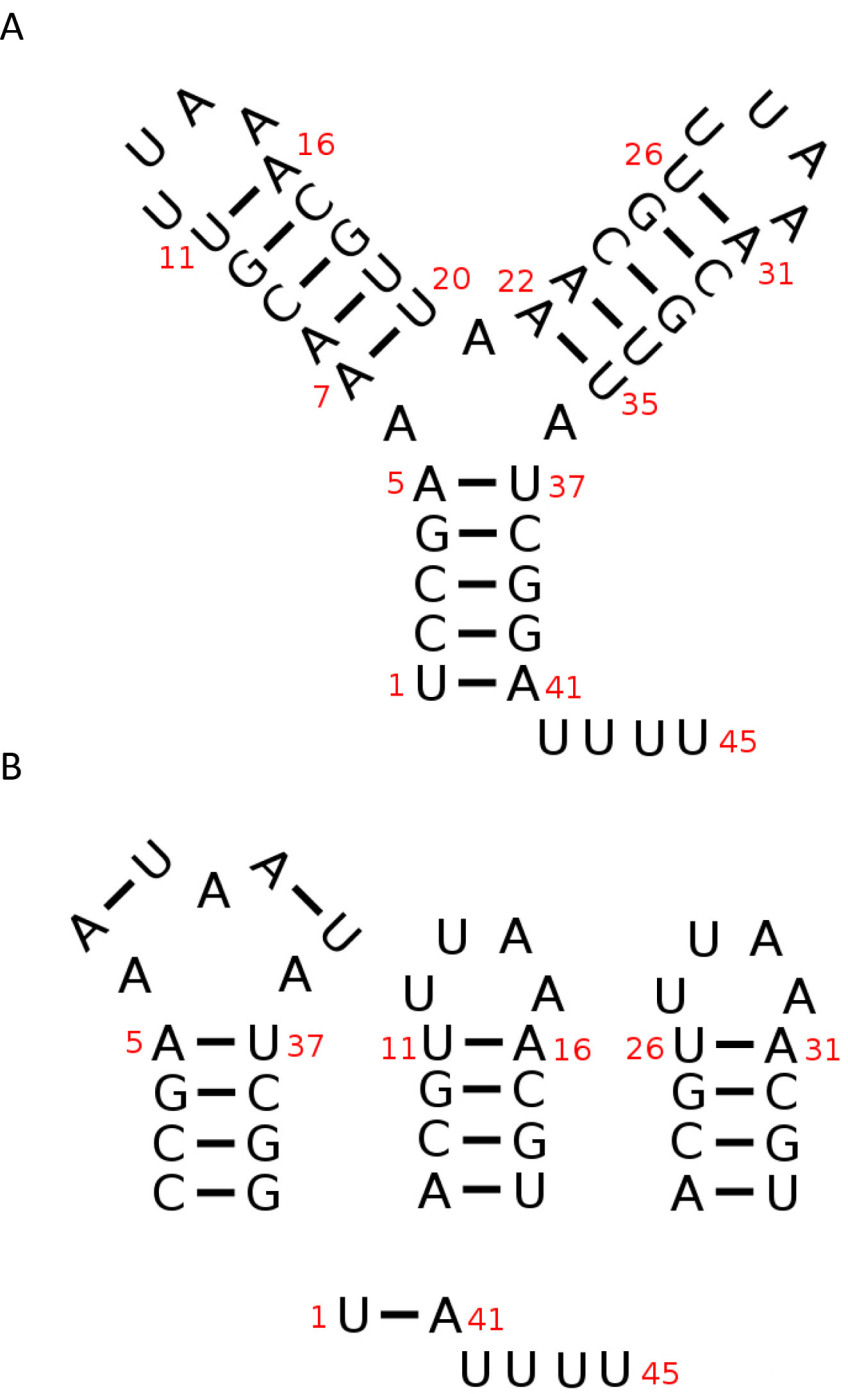
Illustration of the loop elements of an RNA. (**A**) Secondary structure of a 45 nt RNA with various nucleotides labelled by their position in red. (**B**) The RNA secondary structure can be decomposed into four loop elements labelled by the number pairs *L*_1_ = (5, 37), *L*_2_ = (11, 16),*L*_3_ = (26, 31) and *L*_4_ = (45, 1). Note that *L*_4_ indicates the external loop of the RNA.

The KFOLD algorithm represents a secondary structure of an RNA (Figure 1) by a series of smaller structural elements which contain a loop of single-stranded nucleotides containing interspersed single base pairs and a continuous Watson-Crick (WC) helix at the closing base pair of the loop. For simplicity purposes, these small structural elements, illustrated in Figure 1B, will be referred to as loop elements and denoted by the symbol *L*. The loop element can be characterized by the number of single WC base-pair elements in the loop, also called its degree *d* (25), which denotes the number of WC base pairs bordering the loop. Loop elements with *d* = 1 are hairpin turns and contain a series of single-stranded bases closed by a WC helix. A *d* = 2 loop is either a bulge, or internal loop, while *d* > 2 loops are multi-branch loops. Any RNA structure can be decomposed into a unique set of loop elements. In KFOLD, each loop element *L*_*l*_ is labelled by the closing base-pair (*i, j*) with *i* < *j* of the loop which begin/end the loop. The sole exception to this rule is the special external loop which contains the first and last nucleotides and is labelled by (*N*, 1). The inversion (*j, i*) allows the KFOLD programme to distinguish external loops from internal loops in the rare case when the last nucleotide of a sequence is base-paired to the first nucleotide by a lonely base pair. In this case, there will be both an internal loop labelled by (1, *N*) and an external loop labelled by (*N*, 1). Figure 1 shows an example of the loop elements for an RNA containing 45 nt. The secondary structure of this RNA has *N*_*L*_ = 4 loop elements, as illustrated in Figure 1B, with number pairs given by

(3) \begin{eqnarray*} L_1 &=& (5,37),\quad L_2 = (11,16),\quad L_3 = (26,31),\nonumber \\ L_4 &=& (45,1). \end{eqnarray*}

Note that the loop element *L*_4_ = (45, 1) = (*N*, 1) labels the special external loop which contains the nucleotides {1, 41, 42, 43, 44, 45}.

**Figure 2.**
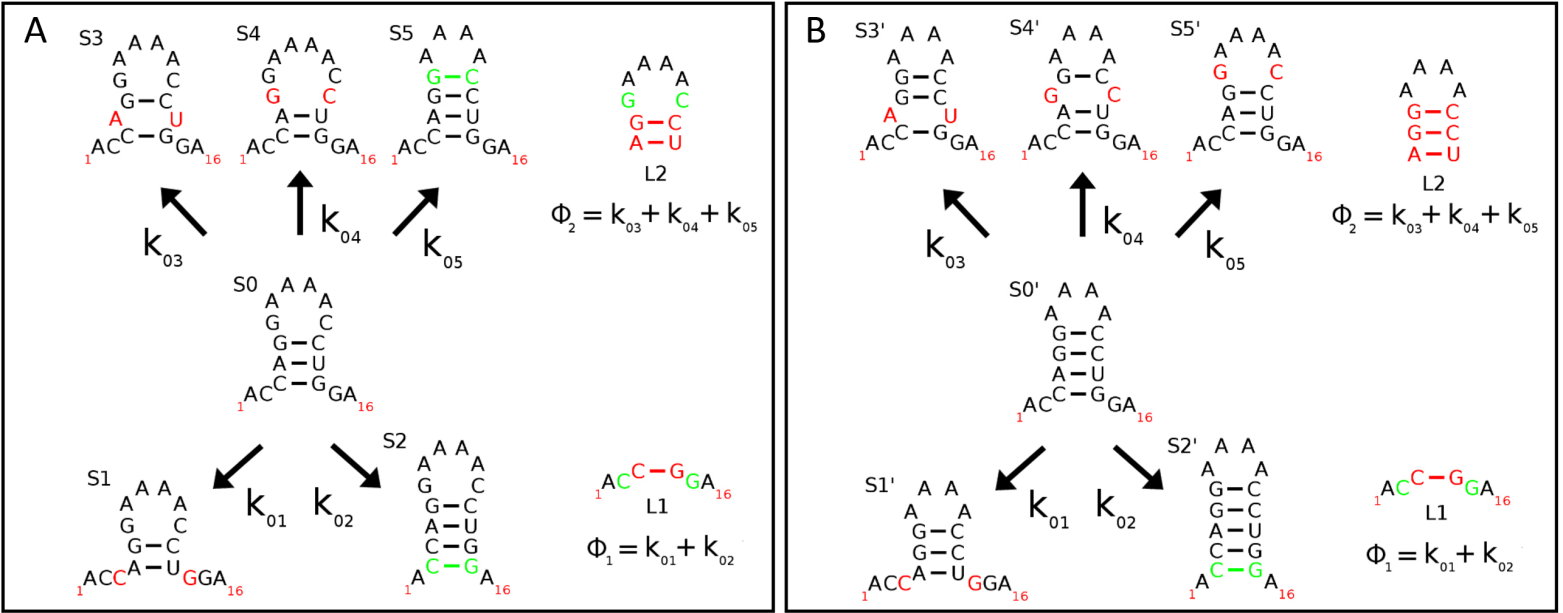
Diagram illustrating the calculation of neighbouring RNA structures by loop element. Nucleotides highlighted green indicate reactions in which a base pair can be added to the current structure while those highlighted red indicate reactions in which a base pair can be removed. (**A**) Pictorial representation of the five neighbours of *S*_0_. In a Gillespie procedure for RNA folding, *S*_0_ will transition to one of the structures *S*_*i*_ in the next reaction. Transition rates, *k*_0*i*_, are shown for each of the reactions. The five possible reactions can be partitioned by the two loop elements *L*_1_ and *L*_2_ and partial fluxes ϕ_1_ and ϕ_2_ computed for each. (**B**) Pictorial representation of the five neighbours of $S^{\prime }_0$. Note, the reactions for loop element *L*_1_ are identical to those in (A) and thus the transition rates for $S^{\prime }_0 \rightarrow S^{\prime }_1$ and $S^{\prime }_0 \rightarrow S^{\prime }_2$ will be identical to those for *S*_0_ → *S*_1_ and *S*_0_ → *S*_2_. Thus, in the event of a transition from $S_0 \rightarrow S_5 = S^{\prime }_0$, the partial flux ϕ_1_ does not need to be recalculated.

The KFOLD algorithm breaks up the neighbour problem by computing neighbouring structures of the loop elements which make up the current RNA fold and storing the portion of the total transition flux that is due to possible transitions to these neighbours. Figure 2 illustrates an example of the partitioning of neighbour structures for a small hairpin with five neighbour structures. The structure *S*_0_ represents the current RNA secondary structure while *S*_*i*_ with *i* = {1, 5} represent the neighbours. Two neighbours (*S*_1_ and *S*_2_) can be associated with loop *L*_1_, while three (*S*_3_, *S*_4_ and *S*_5_) can be associated with loop *L*_2_ as illustrated in Figure 2A. One can see that the difference between, for example, the structures *S*_1_ and *S*_2_ associated with loop element *L*_1_ and *S*_0_ is a base-pair addition/deletion in loop *L*_1_. In addition to breaking up neighbour computations, the KFOLD algorithm will also break up the computation of the total flux Φ by loop elements. For the example shown in Figure 2, the KFOLD algorithm would compute two *partial* fluxes ϕ_1_ = *k*_01_ + *k*_02_ and ϕ_2_ = *k*_03_ + *k*_04_ + *k*_05_, and store these for later use in computing the total flux Φ = ϕ_1_ + ϕ_2_.

There is a substantial time advantage to computing neighbouring structures by the local secondary structural elements of the RNA and storing the partial flux ϕ_*l*_ associated with it as illustrated in Figure 2. This is because when a new structure *S*_*i*_ is selected during the Gillespie algorithm (i.e. *S*_0_ will transition to *S*_*i*_), new neighbouring structures of *S*_*i*_ can be computed by looking at the base-pair additions/deletions that can occur in individual loop elements. The computational advantage stems from the fact that for the vast majority of loop elements in the RNA (i) the total number of reactions for the loop element, (ii) the transition rate for those reactions and (iii) the contribution to the total flux from that loop element, ϕ_*l*_, will remain *unchanged*. Thus, one only needs to calculate new base-pair moves for a few (at most three) of the loop elements in the entire RNA structure after each secondary structure update. Figure 2 illustrates how the partial flux for loop element *L*_1_ would not need to be recalculated when the structure *S*_0_, shown in Figure 2A, transitions to structure *S*_5_ (note $S_5=S^{\prime }_0$ in Figure 2B). Comparing Figure 2A and B, one can see that the base-pair additions/deletions possible for loop *L*_1_ and their respective transition rates are identical.

**Figure 3.**
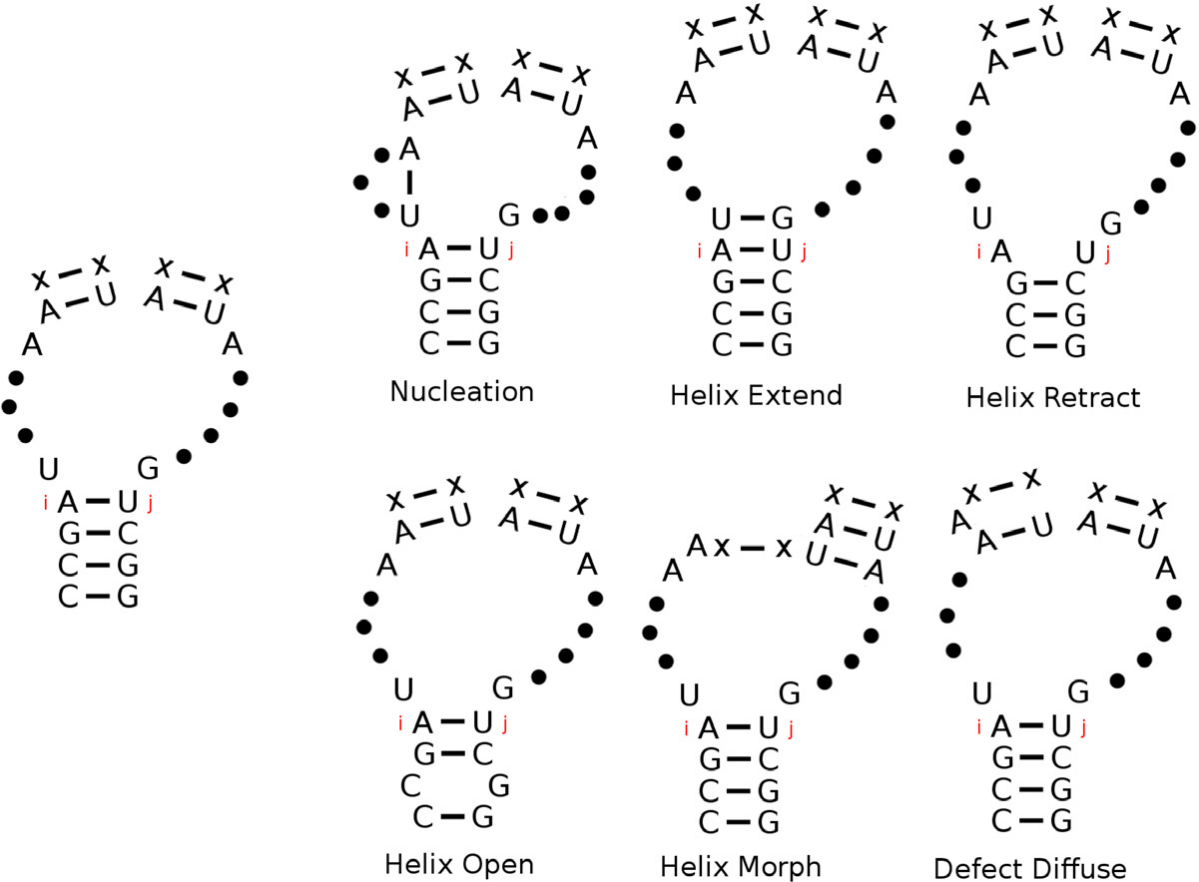
Illustration of the move set used for computing neighbours of a loop element in KFOLD. The six moves that are allowed are (i) helix nucleation; (ii) extension of a base pair at the end of a helix; (iii) retraction of a base pair at the end of a helix; (iv) opening of base pairs within the terminal helix which closes the loop; (v) helix morphing; and (vi) defect diffusion of a single nucleotide.

Although only reactions involving base-pair opening and base-pair formation are illustrated in Figure 2, it is possible to incorporate more complex reactions into the KFOLD algorithm with negligible computational cost and without changing the logarithmic scaling of the algorithm. Figure 3 illustrates the six types of moves (the move set) that can occur in each loop element and that are currently modelled in KFOLD: (i) nucleation; (ii) helix extension; and (iii) helix retraction along one of the *d* base pairs in the loop; (iv) opening of a base pair within the terminal helix; (v) helix morphing; and (vi) defect diffusion. The move set is the same as previously used by Flamm (11), with the one exception that multiple nucleotides cannot diffuse into a helix in a single move (c.f. Figure 3). Also note that this move set will satisfy detailed balance, i.e. each reaction has a backwards reaction with the rates satisfying Equation (1). Pseudoknot reactions involve the nucleation of single-stranded nucleotides in one loop with those in another loop and are currently not modelled in KFOLD. It should be possible to incorporate some types of simple pseudoknot reactions into KFOLD in the future by looking at loops separated by a single helix. Reaction rates could be estimated using similar techniques employed by KINEFOLD (15,16). Long distance pseudoknots are potentially more difficult to incorporate because Gillespie models have no information about the spacial geometry of the fold and potential steric clashes which would prohibit some long-distance pseudoknots from forming.

With this move set and strategy for computing neighbours, it can be shown that the computational cost to compute the partial flux and neighbours of a loop element appears to perform logarithmically over a long simulation (see Supplementary Table S1), while the growth for the total computational cost per Gillespie update is bounded by Log(*N*) (c.f. Supplementary Figure S1). Since new neighbours only need to be recomputed for a couple of loop element after each update of the secondary structure, the total cost of updating the data structure of neighbours in the KFOLD algorithm should perform logarithmically with sequence size when averaged over a large number of steps.

#### Computing the flux and selecting a transition in KFOLD

In addition to determining neighbours, the Gillespie algorithm for RNA folding also requires that the total flux Φ = ∑_*i* = 1, *m*_*k*_0*i*_ be computed, where *k*_0*i*_ are the individual transition rates calculated using Equation (2). A transition can then be selected by finding the first state *S*_*j*_ with transition rate *k*_0*j*_ such that ∑_*i* = 1, *j*_*k*_0*i*_ ≥ *r*Φ, where *r* is a random number in the interval [0, 1]. The simplest way to compute the total flux would be to sum over all the partial fluxes $\Phi = \sum _{l=1,N_L} \phi _l$ which are stored for each loop element. A combinatorics analysis of RNA structures in Hofacker *et al*. (25) showed that, in the limit of large *N*, the number of loops in an RNA secondary structure would scale with sequence size as O(*N*). However, since all but a maximum of three of the partial fluxes ϕ_*l*_ for each of the loop elements in an RNA structure will remain *unchanged* between Gillespie steps, a partial sum table can be used to reduce the computational cost of calculating the total flux Φ and selecting a transition to O(Log_2_*N*). Details of the partial sum table and how a specific transition is chosen can be found in the supplementary material with a specific example of the partial sum data structure illustrated in Supplementary Figure S2. The computational cost of calculating the total flux is demonstrated in Supplementary Figure/Table S1.

#### KFOLD pseudo-code

A generic description of the pseudo-code for the KFOLD algorithm is now given. Note that the pseudo-code makes no assumptions about the move set; i.e. the move set can be chosen to be the six moves shown in Figure 3 or something more complicated (such as reactions involving pseudoknot formation). With these caveats, the basic KFOLD procedure can be given as follows:

1. Extract the total flux Φ from the partial sum table.
2. Choose two random numbers *r*_1_ and *r*_2_ on the interval [0, 1].
3. Increment the time by τ = −ln(*r*_2_)/Φ.
4. Identify the first loop element *L*_*i*_ which satisfies ∑_*l* = 1, *i*_ϕ_*l*_ ≥ *r*_1_Φ by using the partial sum table. During the identification of *L*_*i*_, calculate the remainder $\bar{\Phi } = r_1\Phi - \sum _{l=1,i} \phi _l$ on the fly.
5. Out of the possible transitions in the loop *L*_*i*_ which are determined by the move set, choose a transition *S*_0_ → *S*_μ_ to fire by summing over the transition rates *k*_0*j*_ for each of the base-pair reactions that are possible for this loop until $\sum _{j=1,\mu } k_{0j} \ge \bar{\Phi }$.
6. Move the structure to state *S*_μ_.
7. If the move to state *S*_μ_ results in loops being created (such as during nucleation) or destroyed (such as when two helices merge), add or subtract these loops from the list of loop elements.
8. Re-calculate reactions, transition rates and the partial flux ϕ_*i*_ for loop *L*_*i*_ according to the move set as well as for any other loops that where created. Re-sum the partial sum table after the partial flux for each loop has been re-calculated. Goto STEP 1.

Supplementary Figure S2 illustrates the partial sum table and the process of choosing a reaction to fire in the KFOLD algorithm for the example transition *S*_0_ → *S*_5_ shown in Figure 2. Note that although the modified process of choosing a reaction in KFOLD involves first choosing a loop, followed by choosing a specific reaction in that loop, only a single reaction will end up being chosen from the full set of *m* possible ones. Supplementary Figure S1 demonstrates the runtime and performance of the KFOLD code. As can be seen in Supplementary Table S1, the cost of computing a Gillespie update in the KFOLD algorithm is expected to perform logarithmically according to the mathematical analysis - c.f. Supplementary Figure S1. The runtime analysis also shows that KFOLD can compute 10^7^ RNA folding steps on large RNAs substantially faster than KINFOLD and in a similar time frame to small RNAs, while KINFOLD scales as O(*N*) for large *N* as expected.

#### Availability

The KFOLD program is written in FORTRAN and the source code is available from GITHUB at https://github.com/edykeman/kfold or from the author's personal website at http://www-users.york.ac.uk/∼ecd502/.

## RESULTS AND DISCUSSION

KFOLD is applied to two different RNA folding examples with different lengths. The first example is of a small 20 nt synthetic sequence while the second is the 56 nt RNA from the spliced leader of *Leptomonas collosoma*. These two examples provide a simple illustration of the KFOLD algorithm and are used to benchmark its accuracy. The energy model used for the calculations is the Turner 99 energy model (1) which contains terms for dangle energies, terminal mismatches, and a logarithmic penalty formula for multi-loops. No coaxial stacking energies are currently incorporated, but the energy functions in KFOLD are modular and additional terms such as these can be incorporated. Nucleation rates in KFOLD are obtained from polymer theory formulas (26–28). Supplementary Figure S3 illustrates the behaviour of the forward rate of base-pair nucleation used in KFOLD as a function of nucleotide length. Reaction rates for the remaining moves (ii)-(vi) shown in Figure 3 are calculated using the Kawasaki formula (see Equation (2)), with pre-factors estimated from experimental and theoretical work (29–31). Specific details about the choice of pre-factor used to calculate the transition rates *k*_0*i*_ can be found in the supplementary material. Note that since KFOLD uses real estimates for the kinetic rates the simulations times discussed correspond to real experimental time estimates.4

**Figure 4.**
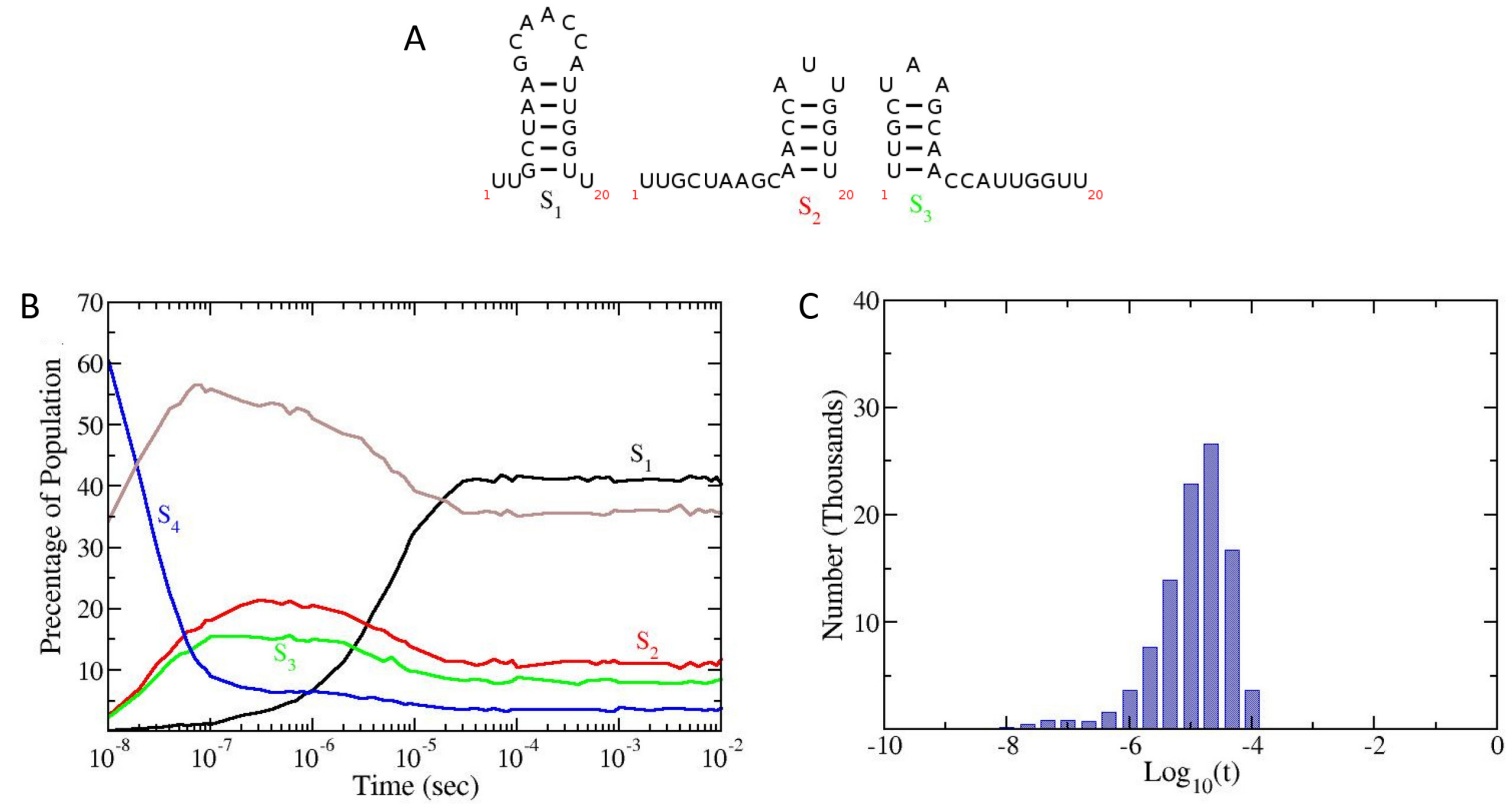
Folding kinetics of a small 20 nt RNA sequence computed with the KFOLD algorithm. A total of 10^5^ folding simulations were generated for the sequence UUGCUAAGCA ACCAUUGGUU. The three lowest energy structures are monitored along with the denatured state. Each simulation computed the folding trajectory of the RNA and output the structure at regular time intervals up to a maximum time of 10 ms. (**A**) The three lowest energy structures are labelled S_1_ (lowest energy structure) to *S*_3_. (**B**) Population kinetics for the three lowest energy structures. The open chain configuration *S*_4_ is shown in blue and the brown line represents the population of all other structures which are not *S*_1_–*S*_4_. (**C**) Histogram of the first passage times for the RNA to fold from the completely denatured state to the MFE structure *S*_1_. The base 10 log of the first passage times are used to create the plot.

### Folding kinetics of a small 20 nt RNA

**Figure 5.**
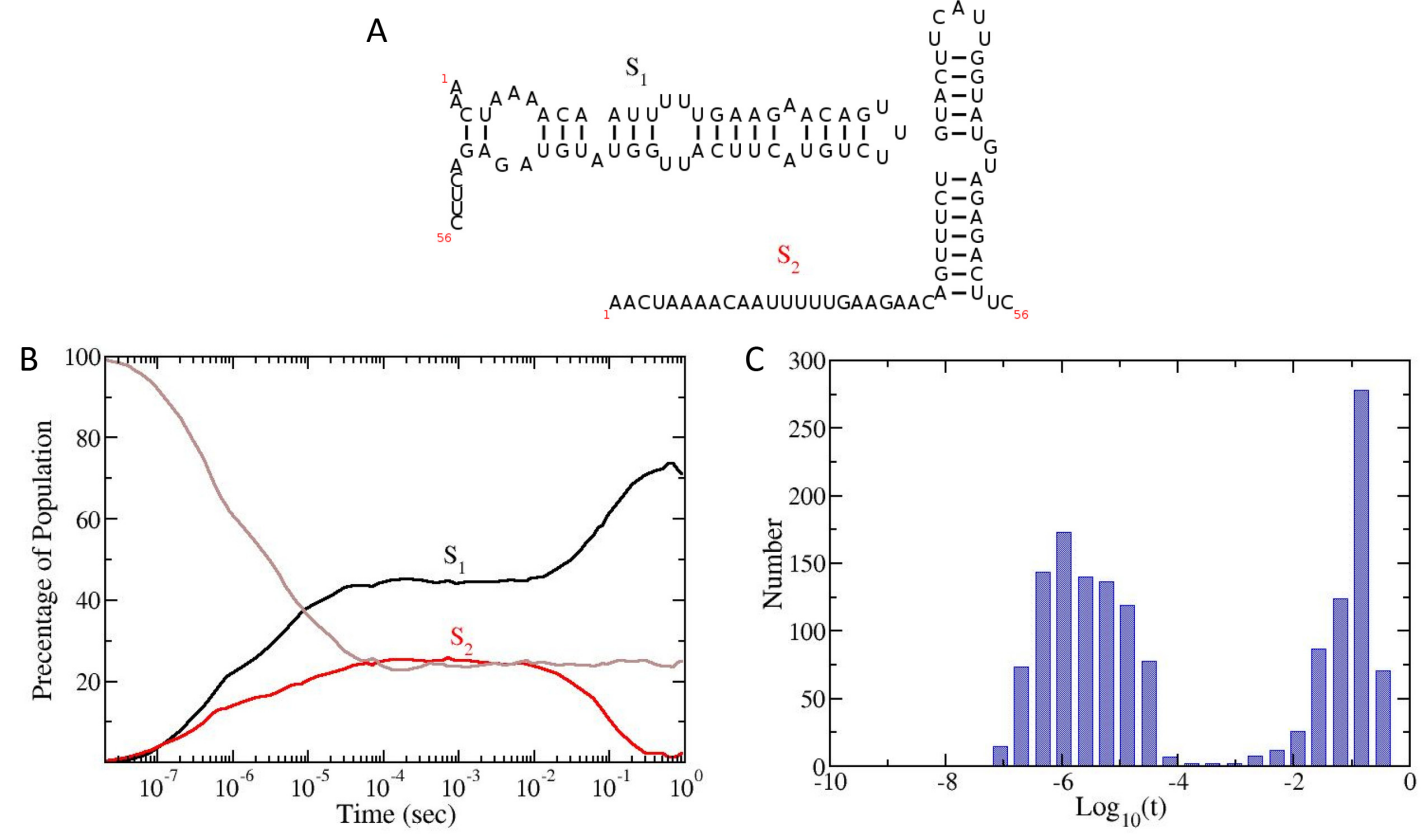
RNA folding kinetics of a small 56 nucleotide spliced leader RNA from Leptomonas collosoma computed with the KFOLD algorithm. (**A**) The two lowest energy structures of the spliced leader RNA predicted using MFOLD algorithm (2) which uses the Turner 99 energy rules. The structures are labelled *S*_1_ (lowest energy structure) and *S*_2_. (**B**) Folding kinetics of the spliced leader RNA predicted using KFOLD. A total of 1500 separate trajectories were computed and the number of RNAs within the ensemble having either structure *S*_1_ or *S*_2_ calculated. The unlabelled brown line indicates all other folds which were not in either structure *S*_1_ or *S*_2_. (**C**) Histogram of the first passage times for the RNA to fold from the completely denatured state to the MFE structure *S*_1_. The base 10 log of the first passage times are used to create the plot.

To test the accuracy of the KFOLD algorithm and insure that it reproduces the equilibrium thermodynamics of an RNA at long times, the folding kinetics of the small 20nt RNA sequence UUGCUAAGCA ACCAUUGGUU was calculated. Using RNAsubopt from the ViennaRNA package, all possible structures satisfying canonical (G-C, A-U, G-U) base-pairing were calculated. The RNAsubopt program found 4127 different RNA states for the 20nt sequence. The energy of these structures were then computed using the Turner 99 energy model and the partition function calculated along with the probability of each state at thermal equilibrium. A total of 10^5^ folding trajectories were generated using KFOLD, with each trajectory simulating folding up to a maximum time of 10 ms (note this is roughly equivalent to setting the maximum simulation time in KINFOLD between 10^7^ to 10^8^). Figure 5B shows the probability of occurrence of the three lowest energy states in the ensemble (c.f. Figure 5A) along with the unfolded state *S*_4_ as a function of time. As can be seen from the graph, KFOLD reproduces the correct equilibrium populations predicted using the partition function (i.e. *S*_1_ = 41.3%, *S*_2_ = 11.3%, *S*_3_ = 8.2%).

In addition to calculating population kinetics, the first passage times for the RNA to fold from the completely denatured state to the MFE state (structure *S*_1_) were also calculated. Figure 5C shows a histogram of the folding times. The times used to generate the histogram in Figure 5C are the base 10 Log of the folding time in seconds. This reveals an average time of around 10μs for the first time the RNA will encounter the MFE state. Both of these results are consistent with mean first passage times generated from the KINFOLD program (when KINFOLD times are multiplied by a constant factor), which is to be expected since KFOLD performs the same Gillespie procedure for RNA folding, just in a more efficient way.

### Folding kinetics of the spliced leader from *Leptomonas collosoma*

The spliced leader RNA from *Leptomonas collosoma* is a 56-nt RNA which adopts two different stem-loop structures (32) and is present in mature mRNAs of trypanosomatid protozoa. Figure A illustrates the two lowest RNA secondary structure states predicted using the Turner 99 energy model. The KFOLD algorithm was used to compute 1500 separate folding trajectories of the RNA sequence starting from the completely denatured state. A total of 1 s of folding was computed along with the first passage times for the RNA to fold into the MFE state (state *S*_1_).

As can be seen in Figure B, the RNA folds predominantly into either structure *S*_1_ or *S*_2_. Interestingly at around 100 ms, the structure *S*_2_ decays into the MFE structure, suggesting that *S*_2_ forms a temporary kinetic trap. This is further supported by the first passage times shown in Figure C. Here, one can see that there are two peaks in the distribution of folding times, one at around 1 μs and the other at around 100 ms. In combination with the kinetic trajectory shown in Figure B, this data suggest that there are two pathways to fold to the MFE state. The first pathway is fast and takes the structure directly into the MFE state. The second is a slow pathway which is interrupted by a temporary kinetic trap, the state *S*_2_. The time to resolve this kinetic trap and fold into the MFE state is roughly 100 ms. These results roughly match *in vitro* experiments (32) which show the predominant fold of the RNA *ex vivo* in the absence of proteins is *S*_1_. It should be noted again that the energy model used in this simulation is the Turner 99 energy model, not the 2004 model. Other groups have folded the same RNA sequence using the Turner 2004 model and have obtained different folding kinetics for the RNA (13).

## CONCLUSION

Fast, efficient and accurate RNA secondary structure prediction and RNA folding kinetics algorithms remain one of the foremost challenges in computational biology. Gillespie-type models for RNA folding kinetics have suffered from two major drawbacks: (i) that the computational cost to compute a fixed number of changes to the secondary structure of the RNA scales with the number of secondary structure neighbours *m* which can be as high as *N*^2^; and (ii) that many trajectories of the RNA are required to be computed in order to obtain reasonable statistics of the kinetics. The KFOLD algorithm addresses one of these issues by presenting an improvement to the scaling and performance of Gillespie-type RNA kinetics algorithms which model RNA folding in single base-pair addition/deletion resolution. As demonstrated above, the improved algorithm performs logarithmically with sequence size as opposed to the O(*N*)-O(*N*^2^) scaling of the traditional method. It should be noted that although KFOLD can compute updates to the secondary structure quickly, it still suffers from the remaining problem shared by all Gillespie-type folding models, i.e. the requirement for a large number of trajectories to compute accurate statistics. However, by speeding up the computation of RNA folding trajectories as demonstrated here, KFOLD should be capable of generating many more folding trajectories in the same CPU time as the traditional Gillespie procedure, thus reducing the overall time to generate the folding statistics for an RNA.

It is expected that through the use of additional computational techniques, such as coarse graining of RNA restructuring, memorization (21) or other Gillespie techniques (33), to improve the efficiency, further reductions in time could be potentially achieved. Moreover, the KFOLD algorithm has the potential to incorporate pseudoknot reactions with limited cost. With these further improvements, Gillespie-type models have the potential of being applied to more complex RNA kinetics problems.

## SUPPLEMENTARY DATA

Supplementary Data are available at NAR Online.

SUPPLEMENTARY DATA
