# Supplementary material for: An implementation of the Gillespie algorithm for RNA kinetics with logarithmic time update
Source: Nucleic Acids Res. 2015 May 18;43(12):5708–15. doi: 10.1093/nar/gkv480 (PMC4499123; doi:10.1093/nar/gkv480)
Supplement: SUPPLEMENTARY DATA [file supp_43_12_5708__index.html]

An implementation of the Gillespie algorithm for RNA kinetics with logarithmic time update — An implementation of the Gillespie algorithm for RNA kinetics with logarithmic time update — SUPPLEMENTARY DATA 

# An implementation of the Gillespie algorithm for RNA kinetics with logarithmic time update

## SUPPLEMENTARY DATA

- SUPPLEMENTARY DATA
