# Supplementary material for: An implementation of the Gillespie algorithm for RNA kinetics with logarithmic time update
Source: Nucleic Acids Res. 2015 May 18;43(12):5708–15. doi: 10.1093/nar/gkv480 (PMC4499123; doi:10.1093/nar/gkv480)
Supplement: SUPPLEMENTARY DATA [file supp_gkv480_nar-00767-z-2015-File004.pdf]

1 Runtime and Scaling of KFOLD

To demonstrate the running time and scaling of the KFOLD algorithm, KFOLD was used to compute a total of 10^5 single base-pair additions or deletions (or equivalently 10^5 Gillespie steps) on 100 RNA sequences of varying sequence composition. RNA lengths of 20, 40, 80, 200, 400, 800 $\cdots$ up to 8000 nucleotides were used to benchmark the runtime. For each sequence length, the RNA was started from the minimum free energy (MFE) structure and 10^5 folding steps performed for each of the 100 different RNA sequences. KFOLD was profiled using the gnu profiler to separate the cost of computing neighbours from the cost of computing the total flux, which are done by separate subroutines.

Using the same RNA sequences used in KFOLD, the running time to compute 10^5 base-pair additions/deletions for the 100 RNA sequences was also calculated for KINFOLD, again starting from the MFE structure. Timing of KINFOLD was done using the gnu time function. Source code for the KINFOLD program was obtained from ViennaRNA2.1.8 and modified to perform precisely 10^5 steps then stop. KINFOLD was executed with the following settings: –start –stop –silent –num 1 –time 10^{10}. The starting structure was the same minimum free energy structure used in KFOLD while the stop structure was set to the open chain. For KINFOLD run times over 2000 seconds, a smaller number of Gillespie steps where performed and the run time scaled appropriately.

Supplementary Figure 1 illustrates the scaling of the KFOLD and KINFOLD algorithms, while Table 1 gives the specific data plotted. As can be seen from the figure, KFOLD performs a fixed number of Gillespie updates in the RNA folding problem with logarithmic scaling while KINFOLD scales roughly linearly at large N.

2 Partial Sum Table and Transition Selection

The KFOLD algorithm uses a partial sum table to compute the total flux Φ as well as select an individual transition to fire. The total flux is the sum over all the m possible transition rates to neighbouring structures

$$\Phi = \sum_{i=1}^{m} k_{0i} \tag{1}$$

and is needed in the Gillespie algorithm in order to sample the individual transitions with the appropriate probability. In KFOLD, neighbours are

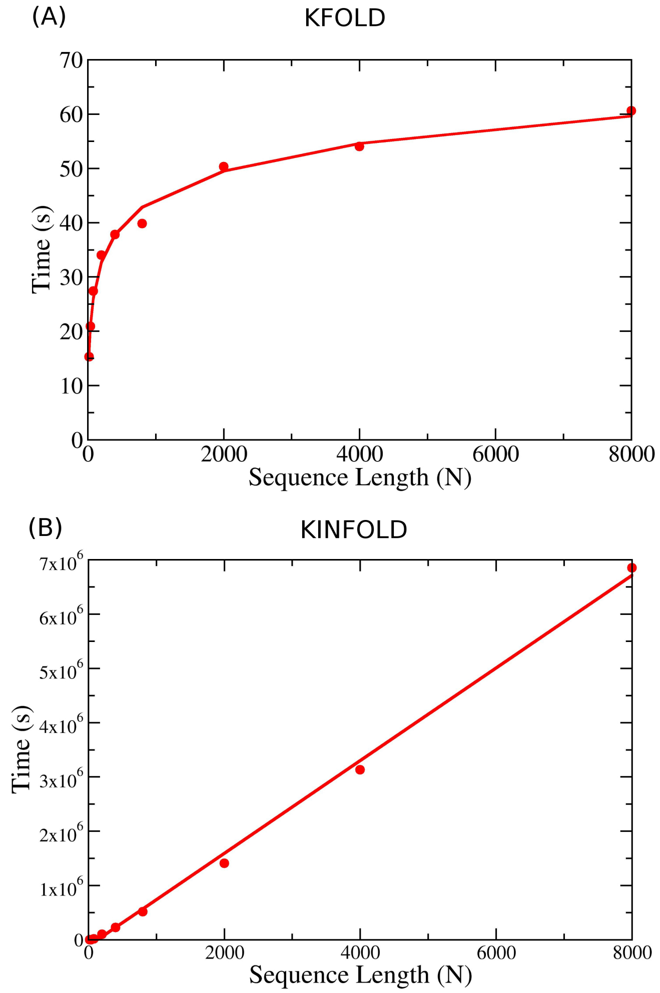

Supplementary Figure 1: Approximate scaling of the KFOLD and KINFOLD algorithms. Data points are shown as red dots and are the run times to compute 10^5 Gillespie steps on 100 RNA sequences for different sequence lengths. **(A)** Plot of the computational run time for KFOLD. The solid red line illustrates the best fit of the logarithmic function $T = a{*}\text{Log}(N) + b$ to the data points where a and b are constants. The correlation coefficient for this fit is 0.9959. **(B)** Plot of the computational run time for KINFOLD. The solid red line illustrates the best fit of the linear function $T = a * N + b$ to the data points where a and b are constants. The correlation coefficient for this fit is 0.9946.

Supplementary Table 1: Comparison of the approximate run times of KFOLD and KINFOLD. For each sequence length, both algorithms were used to compute 10^5 Gillespie steps on 100 RNAs with different sequence composition. The table gives the total run time to compute 10^5 steps for all 100 sequences for a total of 10^7 steps. Calculations were performed on a single 2.8 GHz Intel Xenon processor with 12GB of RAM. a) Approximate time devoted to computing the total flux Φ using the partial sum table. b) Approximate time devoted to computing neighbours and partial fluxes of a loop element. *Indicates that the KINFOLD calculation used the –noShift setting to remove the calculation of defect diffusion reactions.

	Φ^a	Neighbors^b	KFOLD	KINFOLD*	KINFOLD
L(nt)	Time(s)	Time(s)	Time(s)	Time(s)	Time(s)
20	6.20E-01	1.46E+01	1.53E+01	1.08E+01	7.52E+01
40	5.50E-01	2.03E+01	2.09E+01	5.89E+01	1.07E+03
80	1.05E+00	2.63E+01	2.74E+01	2.62E+02	1.53E+04
200	1.57E+00	3.24E+01	3.40E+01	1.35E+03	1.02E+05
400	1.94E+00	3.59E+01	3.78E+01	1.86E+03	2.26E+05
800	2.24E+00	3.74E+01	3.98E+01	2.63E+03	5.15E+05
2000	2.67E+00	4.76E+01	5.03E+01	4.43E+03	1.41E+06
4000	3.27E+00	5.06E+01	5.40E+01	9.08E+03	3.13E+06
8000	4.07E+00	5.63E+01	6.06E+01	2.57E+04	6.85E+06

partitioned by loop element L_i and the partial flux ϕ_l is stored for each of the N_L loop elements. This partial flux is the sum of Boltzmann factors for each of the reactions that are possible in that loop. This allows the total flux to be written in terms of the partial fluxes as

$$\Phi = \sum_{l=1}^{N_L} \phi_l. \qquad (2)$$

Figure 2 in the main text illustrates how the partial fluxes would work for a small hairpin. For example, the partial flux ϕ_1 is a sum of the transition rates k_{01} and k_{02} (the two reactions that can occur in loop L_1) while ϕ_2 is a sum of the transition rates k_{03}, k_{04}, and k_{05}. Summing gives the total flux Φ which is the sum of all transition rates k_{0i} with $i = [1, m]$. Since the number of loops scales with sequence size N as $\mathrm{O}(N)$, the computational cost of performing the summation in Eq. 2, which is required for computing the total flux Φ in the Gillespie algorithm for RNA folding, is expected to scale as $\mathrm{O}(N)$ in time.

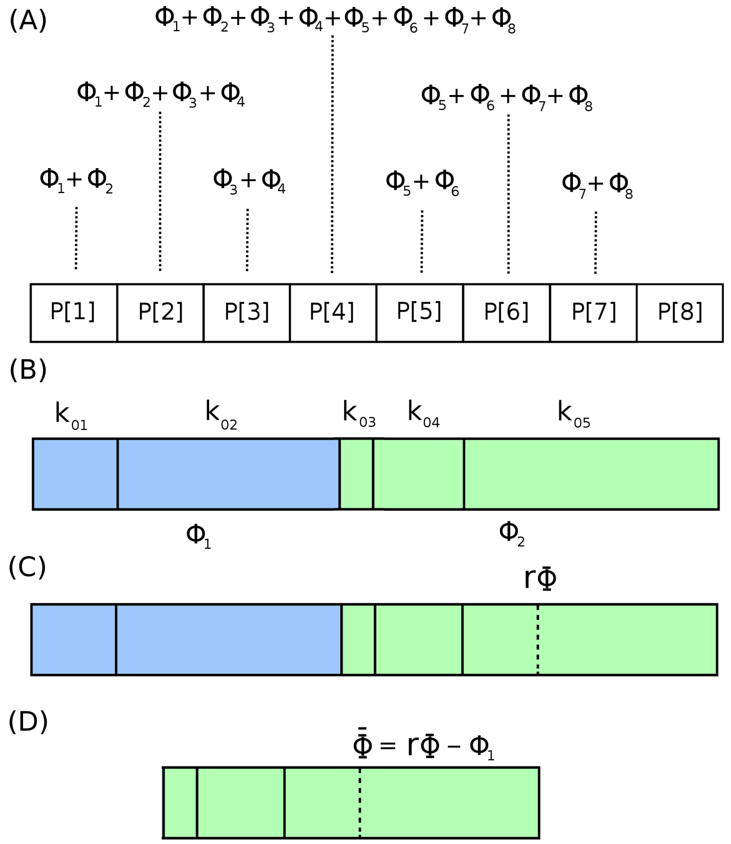

Supplementary Figure 2: Diagram illustrating the partial sum table used in KFOLD. **(A)** Example of the partial sum data structure for the case $N_L = 8$. **(B)** Example of how KFOLD identifies a reaction in a loop element to fire. The example is illustrated for the $S_0 \to S_5$ transition shown in Figure 2a. The transition rate for each of the five reactions are represented by boxes where the width is proportional to k_{0i}. The reactions $S_0 \to S_1$ and $S_0 \to S_2$ are both associated with loop L_1 and the partial flux for this loop is $\phi_1 = k_{01} + k_{02}$. The magnitude of ϕ_1 and its contribution to the total flux Φ is shaded blue while the contribution from ϕ_2 is shaded green. **(C)** Illustration of the threshold $r\Phi$ (dashed line) which is used to choose a transition to fire. KFOLD first identifies the loop element j which satisfies Eq. 4 by using the partial sum table. **(D)** After identifying loop L_2 using the partial sum table, KFOLD sums the individual transition rates k_{0i} which contribute to ϕ_2 until the sum is greater than the value of $\bar{\Phi} = r\Phi - \phi_1$. This occurs for k_{05} indicating this is the reaction that will be chosen.

The purpose of the partial sum table is to reduce the computational cost of performing the summation in Eq. 2 from O(N) to O(Log$_2$ N). Consider an array $P[i]$ which has $i = [1, n_s]$ elements, where n_s is the nearest integer greater than or equal to the number of loop elements N_L in the RNA sequence which is also a power of 2. For example, if $N_L = 7$, then the nearest integer greater than 7 which is a power of 2 is $n_s = 8$. In each element of the array, a partial sum of Eq. 2 will be stored according to the formula

$$P[n/2 + nk] = \sum_{l=nk+1}^{n(k+1)} \phi_l \tag{3}$$

where n ranges in powers of 2 up to $n = n_s$ (i.e. $n = 2, 4, 8, 16, \cdots, n_s$) and k runs over positive integers up to $k_{\max} = (n_s/n) - 1$ (i.e. $k = 0, 1, 2, \cdots, n_s/n - 1$). Supplementary Figure 2a illustrates how each of the array elements $P[i]$ would be filled in the case where $N_L = n_s = 8$. Note that the total flux Φ is stored in the $P[n_s/2]$ element of the array. The partial sum table forms a data structure which allows one to: (i) identify the value j such that $\sum_{l=1,j} \phi_l \geq r\Phi$ where r is a random number between 0 and 1, and (ii) recompute the total flux Φ when only one (or a few) of the ϕ_l values change after updating the secondary structure of the RNA in O(Log$_2$ N) time. To see how this would work, consider the first part, i.e. locating the j such that

$$\sum_{l=1,j} \phi_l \geq r\Phi \tag{4}$$

is satisfied. Starting at $P[n_s/2] = P[4] = \Phi$, one would first check if $r\Phi$ is larger then $P[2]$. If it is, then the j value which satisfies Eq. 4 must be between $j = [5, 8]$ and one would descend the partial sum table to the $P[6]$ element and compare $P[5]$ and $P[7]$ with remainder $\bar{\Phi} = r\Phi - P[2]$. This process would repeat until the j value is located, taking a total of Log$_2(n_s)$ steps. During the process of locating j, the remainder $\bar{\Phi} = r\Phi - \sum_{l=1,j-1} \phi_l$ has also been computed which will be needed to locate a specific transition to fire in the loop L_j. This would be done by looking through the individual transitions that are possible for loop element L_j and locating the specific transition μ such that

$$\sum_{i=1,\mu} k_{0i} \geq \bar{\Phi} \tag{5}$$

Supplementary Figure 2b illustrates this process for the case shown in Figure 2a of the main text where there are two loop elements. After the transition is chosen, and the reactions, their transition rates, and the partial flux for

the loop L_j recalculated, then one can begin the re-summation of the total flux by ascending the partial sum table. For example, if $j = 3$ satisfied Eq. 4 above, then a reaction in loop L_3 would fire and the reactions and transition rates would be re-computed altering the value for ϕ_3. Since none of the other flux values change, only elements in the partial sum array that contain the ϕ_3 term need to be recomputed. This can be done by first re-computing $P[3]$, then re-computing $P[2]$ using $P[1]$ and the new $P[3]$, then finally re-computing $P[4] = \Phi$, with the full computation taking a total of $\mathrm{Log}_2(n_s)$ steps.

The pseudo-code for descending the partial sum table can be written as follows:

```
/* Find loop */

n = nsum;
m = n/2;
Phi = P[m];
x = r * Phi;

WHILE ( n > 2 ) {

  n = n / 2;
  i = m - n/2;
  j = m + n/2;

  IF ( P[i] >= x ) {
    m = i;
  }
  ELSE {
    x = x - P[i];
    m = j;
  }
}

/* choose either loop m or m+1 */

IF ( phi[m] >= x ) {
  /* Fire reaction in loop m which satisfies Eq. 5 */
}
ELSE {
```

```
  x = x - phi[m];
  /* Fire reaction in loop m+1 which satisfies Eq. 5 */
}

/* resum */

P[m] = phi[m] + phi[m+1];

WHILE ( n < nsum ) {

  n = 2 * n;
  m = int(m/n) * n  + n/2;

  i = m - n/4;
  j = m + n/4;

  P[n] = P[i] + P[j];

}
```

During the implementation of the KFOLD algorithm, it may be possible to create or destroy a single loop as a result of the addition or deletion of a single base-pair. This will require a further update to the partial sum table to reflect the fact that a loop has been created or destroyed. However it is important to note that this will not alter the log scaling of the procedure. To see this, suppose that there are currently $N_L = 7$ loops and a new loop gets added to the RNA structure by creating a new hairpin loop within loop number 1. Prior to this new loop being added, the partial flux ϕ_8 was set to zero and $P[7] = \phi_7 + \phi_8 = \phi_7$. So after creating the new loop, one must re-calculate the partial flux for loop 1 (where the new loop was created) and also the partial flux for the new loop number 8. Afterwards, the partial sum table must be updated to reflect changes in both ϕ_1 and ϕ_8. This is easily achieved by simply repeating the resum portion of the pseudocode above on the new loop number 8. This will update only the parts of the partial sum table where ϕ_8 is present (i.e. $P[7], P[6]$,and$P[4]$) in $\text{Log}_2 N$ time. Likewise in the case when there are 8 loops present and a loop is deleted (say loop 5), then one only needs to reset the partial flux for loop 5 to $\phi_5 = \phi_8$ move the last loop in the list to loop number 5, set $\phi_8 = 0$ and then resum the partial sum table for loops 5 and 8 using the resum code which can resum the partial sum table in $\text{Log}_2 N$ time.

3 Kinetic Rates in KFOLD

There are many possible procedures for choosing transition rates between neighbouring structures and their choice will have impact on the folding kinetics. Flamm et al. (11,12) discussed at least two different procedures for computing kinetic rates for single base-pair RNA folding kinetics. The first is a symmetric procedure called Kawasaki dynamics in which transition rates between state S_i with free energy G_i and state S_j with free energy G_j are given by:

$$k_{ij} = k_0 e^{-\beta \Delta G_{ij}/2} \tag{6}$$

with $\Delta G_{ij} = G_j - G_i$ and k_0 equal to a constant. The second is based on the Metropolis algorithm where the transition rate is set to $k_{ij} = 1$ if $G_j < G_i$ and $k_{ij} = e^{-\beta \Delta G_{ij}}$ otherwise. An alternative would be to estimate one of the transition rates between states S_i and S_j, either k_{ij} or k_{ji}, and obtain the second rate using

$$\frac{k_{ij}}{k_{ji}} = e^{-\beta \Delta G_{ij}} \tag{7}$$

The advantage of this is that kinetic rates are now on the order of proper time scales of the transition, allowing the time computed during the folding trajectory to be closer to the correct time scale for folding derived from experiments. In addition, nucleation rates between two nucleotides i and j are likely to be diffusion limited, and hence should be roughly constant and independent of sequence. This is since the rate limiting step of hairpin nucleation is likely to be closely approximated by the time required for the two nucleotides to find each other in a volume. Nucleotides closer together should find each other quicker then nucleotides further apart, thus leading to a higher probability for closely spaced nucleotides to nucleate a hairpin loop. A number of classic polymer theory papers using worm-like chain models of a polymer estimate the probability of loop closure events (26,27,28). Toan et al. derive a general formula for loop closure (28), while Kuznetsov et al. use the results from Toan et al. and apply them to a simple model of RNA hairpin folding (29). Supplementary Figure 3 illustrates the predicted nucleation rates in the diffusion limited regime for a single-stranded RNA polymer. Both the Toan et al. theory, used in Kuznetsov et al. kinetic zipper model (29), and the earlier classical work of Schulten et al. (26) are shown here. Finally, base-pair formation at the end of helices has been estimated from experimental work of Porschke (30,31) to be a fast zippering effect that is much faster than hairpin nucleation events. Kuznetsov et al. use a zippering rate between 10^7 and 10^9 per second in their model.

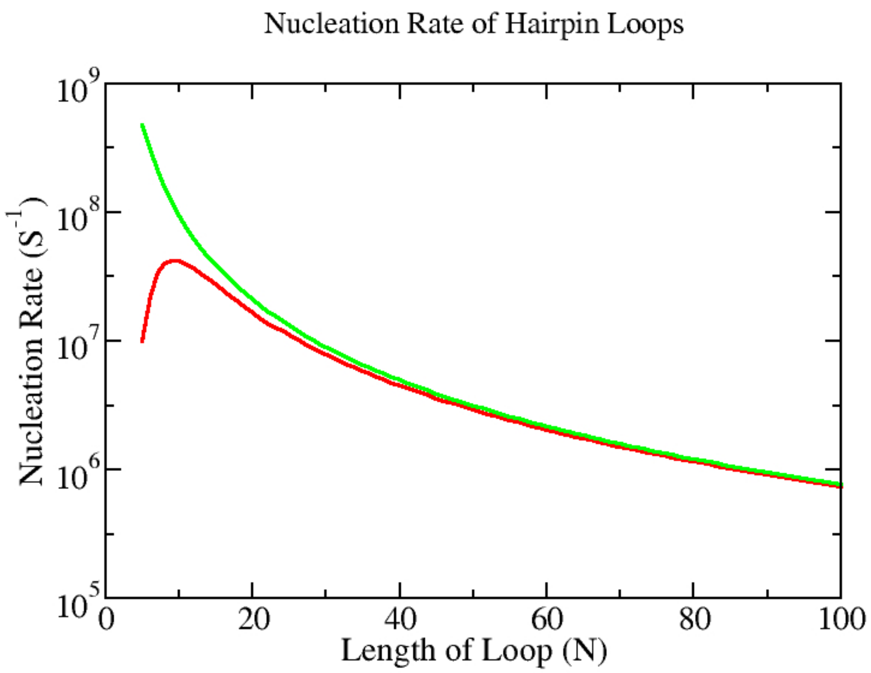

Supplementary Figure 3: Nucleation rate of RNA hairpin loops estimated in the diffusion limited regime from polymer theory. The green line indicates the prediction of Szabo, Schulten and Schulten (SSS) theory in which the nucleation rate scales with the loop length L as $1/L^2$. The red line is the prediction from Toan et al. which is used in this work. The red curve predicts that loops of roughly 10 nucleotides have the fastest nucleation rate.

With these insights into the kinetic rates of RNA folding, the KFOLD algorithm fixes the forward rate for nucleation events using the Toan et al. formula. Backwards rates are then estimated using Eq. 7. Helix extension and helix retraction rates are calculated using the standard Kawasaki formula, i.e.

$$k_{ij} = 10^8 e^{-\beta \Delta G_{ij}/2} \tag{8}$$

where the $k_0 = 10^8$ pre-factor is an estimate for the zippering rate from Kuznetsov (29). The kinetic rates for helix morphing and defect diffusion are also estimated with the same Kawasaki formula, but with a different pre-factor. KFOLD uses the pre-factor of $k_0 = 5 \times 10^6$ for defect diffusion and helix morphing which was obtained from Porschke's experimental work (31), i.e.

$$k_{ij} = 5 \times 10^6 e^{-\beta \Delta G_{ij}/2} \tag{9}$$
